# Transient Localized Wave Patterns and Their Application to Migraine

**DOI:** 10.1186/2190-8567-3-7

**Published:** 2013-05-29

**Authors:** Markus A Dahlem, Thomas M Isele

**Affiliations:** 1 Department of Physics, Humboldt-Universität zu Berlin, Berlin, Germany; 2 Institute of Theoretical Physics, Technische Universität Berlin, Berlin, Germany

## Abstract

**Abstract:**

Transient dynamics is pervasive in the human brain and poses challenging problems both in mathematical tractability and clinical observability. We investigate statistical properties of transient cortical wave patterns with characteristic forms (shape, size, duration) in a canonical reaction-diffusion model with mean field inhibition. The patterns are formed by ghost behavior near a saddle-node bifurcation in which a stable traveling wave (node) collides with its critical nucleation mass (saddle). Similar patterns have been observed with fMRI in migraine. Our results support the controversial idea that waves of cortical spreading depression (SD) have a causal relationship with the headache phase in migraine and, therefore, occur not only in migraine with aura (MA), but also in migraine without aura (MO), i.e., in the two major migraine subtypes. We suggest a congruence between the prevalence of MO and MA with the statistical properties of the traveling waves’ forms according to which two predictions follow: (i) the activation of nociceptive mechanisms relevant for headache is dependent upon a sufficiently large instantaneous affected cortical area; and (ii) the incidence of MA is reflected in the distance to the saddle-node bifurcation. We also observed that the maximal instantaneous affected cortical area is anticorrelated to both SD duration and total affected cortical area, which can explain why the headache is less severe in MA than in MO. Furthermore, the contested notion of MO attacks with silent aura is resolved. We briefly discuss model-based control and means by which neuromodulation techniques may affect pathways of pain formation.

## 1 Introduction

The undoubtedly most fundamental example of transient dynamics is the phenomenon of excitability, that is, all-or-none behavior. Shortly after transient response properties of excitable membranes were classified into two classes [1], it was also explained in a detailed mathematical model how excitability emerges from electrophysiological properties of such membranes in the ground-breaking work by Hodgkin and Huxley [2]. Two features are central and are by no means exclusive to biological membranes but shared by all excitable elements. Firstly, the inevitable threshold in any all-or-none behavior requires nonlinear dynamics. Secondly, the transient response of the system to a super-threshold stimulation eventually has to lead back to a globally stable steady state after some large phase space excursion. This indicates global dynamics, that is, dynamics involving not only fixed points and their local bifurcations but more complex invariant sets, for instance periodic orbits that collide with fixed points. An excitable element is in some sense the washed-up brother of the relaxation oscillator: When the threshold vanishes, a single excitable element usually becomes a simpler behaved—and much longer known—relaxation oscillator [3]. Vice versa, when a saddle-node disrupts a limit cycle and introduces a threshold, the sustained oscillations are reduced to long transient responses after perturbations, that is, the dynamics becomes excitable. In this study, we utilize a similar scenario to disrupt sustained traveling wave solutions in a spatially extended medium such that only transient waves occur. We investigated statistical properties of these transient waves to gain a dynamical understanding of spontaneous episodes in migraine.

We will briefly introduce concepts of excitable elements and excitable media in two-variable reaction-diffusion systems. While we also introduce migraine, the view of migraine as a dynamical disease is more elaborated in the discussion in Sect. 5. A particular focus is set on the idea to introduce an global inhibitory feedback that is also studied in various other systems outside the neurosciences and also in neural field models. Section 2 sets the stage for our canonical model introduced in Sect. 3 from were we proceed to our results on the statistical properties of transient waves in Sect. 4.

## 2 Motivation of a Macroscopic Model for Migraine Aura

### 2.1 Spatiotemporal Behavior of Excitable Systems

An excitable system can either be only time-dependent, which will be called in this study an “excitable element,” or excitable systems can be time- and space-dependent, which we call “excitable medium.” These systems are described by ordinary differential and by partial (integro)differential equations, respectively. The episodic migraine attacks, which we model in this study as transient responses of the cortex described by an excitable medium, remind us more of the transient behavior of excitable elements than of the persistent excited state obtained in excitable media. Therefore, we review the general spatiotemporal behavior of excitable elements and spatially extended excitable media in this section.

Excitability was first described for neurons in the original conductance-based membrane model by Hodgkin and Huxley [2]. This and many more refined versions of neural excitability to date contain four or more dynamic variables, but fortunately this is not essential for excitable systems. In fact, it turned out for excitable elements that the main two classes of excitability are actually amenable to direct analysis in a two-dimensional phase plane by identifying in the conductance-based model fast and slow processes and grouping these into dynamics of just two lump variables [4,5]. Using such a geometrical approach and partly analytical theory, the original empirical classification of excitability was further pursued with bifurcation analysis [6], explaining class I by identifying its threshold as a stable manifold of a saddle point on an invariant cycle and the threshold of class II as a trajectory from which nearby trajectories diverge sharply (called canard trajectory). Extensions to these principal mechanisms involve codimension 2 bifurcations and lead also to bursting in three-variable models, which have been investigated in great detail [7]. However, the two-variable models of a fast activator and slow inhibitor and their phase portraits of class I and II became qualitative prototypes for excitable elements in various biological [8], chemical [9], and physical contexts [10].

**Fig. 1:**
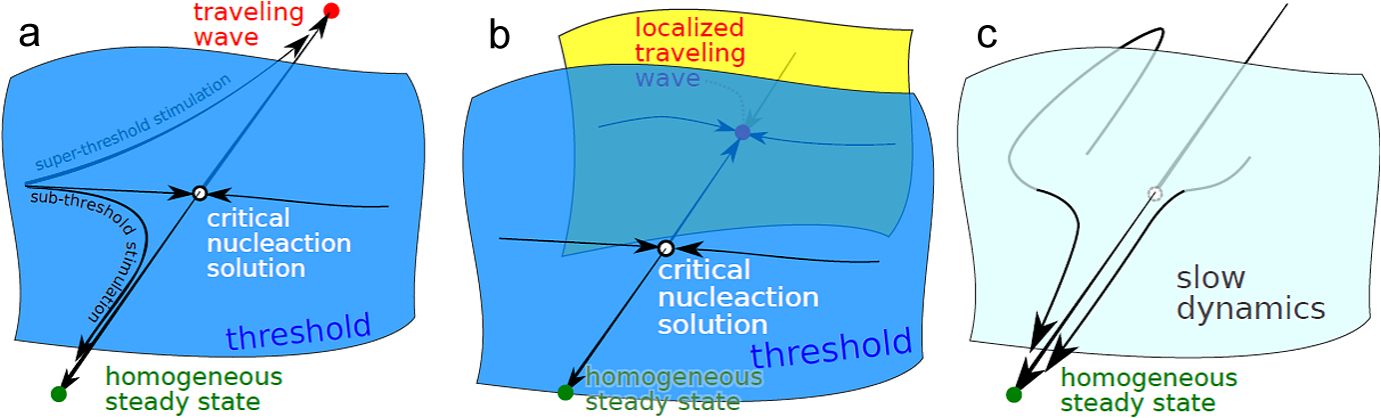
Schematic sketch of the orbit structure in phase space. **a** Excitable media with activator–inhibitor kinetics; the activator diffuses and the inhibitor is immobilized. These systems are multistable, the quiescent state is the homogeneous steady state (*green dot*), and at least one traveling wave solution must exit as the excited state (*red dot*). The basins of attraction between these states are separated by the stable manifold (*blue*) of a critical nucleation solution (*white dot*). Such solutions have only one unstable direction; the corresponding unstable manifold consists of two heteroclinic connections to the stable solutions (*green and red dots*). **b** Excitable media with one activator and two inhibitors, one of which is immobilized, the other fast diffusing or realized by mean field inhibition. The orbit structure in phase space is similar to **a** but the traveling wave solution is now localized similar to the critical nucleation solution to which it is connected. **c** Medium that lost spatial excitability, that is, traveling wave solutions do not exist. Note that the traveling wave solution disappears by a collision of the traveling wave solution with its nucleation solution, that is, in a saddle-node bifurcation. Such systems show ghost behavior, which influences the dynamics in form of *local* excitability (see text)

Distinct from excitable elements and their classification are spatially extended excitable media. Already the original work by Hodgkin and Huxley [2] described spatially extended, tube-like membranes (axons) and introduced the cable equation as a parabolic partial differential equation, which is in the same class as the diffusion equation. In this reaction-diffusion framework, an excitable medium is the continuum limit of a locally coupled chain of excitable elements. Even in reaction-diffusion media with infinite-dimensional phase space, we can again apply geometrical approaches, simply because excitable media are not defined by—in contrast to excitable elements—transient dynamics but traveling wave solutions. The quiescent state is the nonexcited homogeneous steady state. And like the quiescent state, excited states of a medium are usually stationary states in some appropriate comoving frame, for example, along the *x*-direction with ξ=x−ct. Furthermore, the threshold is related to an unstable stationary state, the critical nucleation solution, usually in another comoving frame including c=0. The existence of the nucleation solution is a simple consequence of multistability; see Fig. 1a, but note that even monostable excitable elements have similar unstable stationary states in class I.

In this study, we propose a spatially extended model for wave-like patterns with a characteristic shape, size, and duration. These patterns are (spatially confined) transient responses to confined, spatially structured perturbations of the globally stable homogeneous steady state. These transient responses—after propagating for a while—will always eventually approach the homogeneous state. This leads to a new type of local excitability in a spatially extended medium involving transient traveling wave solutions as invariant sets. Both the model and the initial conditions are motivated by the pathophysiology of migraine and clinical observations [11-13].

### 2.2 Cortical Activity Pattern and Neurovascular Coupling During Migraine

Migraine is characterized by recurrent episodes of head pain, often throbbing and unilateral. In migraine without aura (MO), headache attacks are usually only associated with nausea, vomiting, and sensitivity to light, sound, and even movement [14]. Migraine with aura (MA) involve in addition, but also rarely exclusively, neurologic symptoms (aura) that are caused by waves of cortical spreading depression [12,15-17].

Spreading depression (SD) is a massive but temporary perturbation of ion homeostasis due to seizure-like discharges of neurons. The ion concentrations are usually kept with a narrow range of acceptable limits, while during SD ion concentrations can change by over one order of magnitude to a nearly complete depletion of transmembrane chemical gradients. The ignition of this perturbed ionic balance can spread by diffusion of ions in the extracellular space. Essentially, SD is a slow (about 3 mm/min = 50 μm/sec) reaction-diffusion wave in the approximate 2D cortical sheet of gray matter.

The cortical tissue SD traverses is functionally impaired causing the neurological migraine aura symptoms, like visual hallucinations [11]. Whether SD is also a key to the subsequent headache phase is an open question, in particular, in cases of migraine without aura (MO). If SD occurs in MO, the massive ionic imbalance must remain clinically silent [18,19] or—by definition of diagnostic criteria—neurological symptoms must last less than 5 min.

The aura is usually, though not always [20], before the headache phase and last usually less than one hour [21]. The transient nature of SD (and thus the migraine aura) poses challenging problems in clinical observability. Objective measures by means of noninvasive imaging are in particular difficult to access when clinical symptoms do not indicate the aura phase, i.e., if SD stays silent. Attacks observed with noninvasive imaging are usually triggered, which also could cause a trigger-specific bias. Only one well-documented case of a spontaneous migraine headache supports the still contested notion of “silent aura” [22]: Blood-flow changes where observed that were most likely the result of SD.

SD in the cortex is accompanied by a pronounced increased regional cerebral blood flow for about 2 min and a long lasting (∼2 h) decrease [23]. This naturally raises the question of the physiological relevance of these blood-flow changes. Are they just an epiphenomenon that can be used to indirectly measure SD or do these changes participate in the pattern forming mechanism of SD? We suggest that the initial increase in cerebral blood flow for about 2 min is effectively an inhibitory feedback mechanism for SD. The hyperemic phase (increased blood flow) engulfs large regions of the human cortex [16], while, as we further investigate in this study, the massive ionic imbalance directly due to SD is much more limited in extent [12,13]. This suggests that the inhibitory feedback by neurovascular coupling is a global control mechanism of the neural reaction-diffusion system.

The pattern forming interactions are described in the next section from a mathematical perspective. We end this section by providing some background of the physiological bases of the spatial mismatch between the more globally increased blood flow (hypermia) and more spatially confined ionic imbalance during SD.

**Fig. 2:**
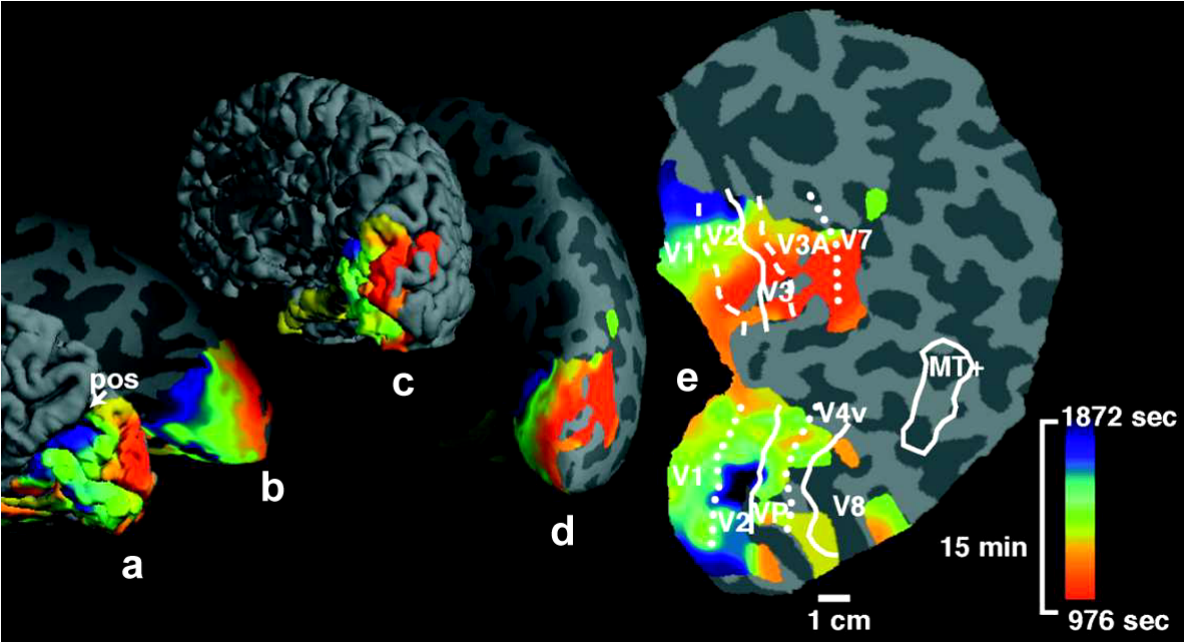
Source localization of the magnetic resonance (MR) data signal of SD (from [12]). Color code: time from onset, locations showing the first MR signals of SD are coded in *red*, later times are coded by *green and blue* (see *color scale to the right*). Signals from the first 975 seconds were not recorded because the migraine attack was triggered outside the MR imaging facility. **a** The data on folded right posterior pole hemispheric cortex; **b** the same data on inflated cortical surface; **c** and **d** the same data shown on the entire hemisphere from posterior-medial view (oblique forward facing), folded and inflated, respectively. As described in the original study [12], MR data were not acquired from the extreme posterior tip of the occipital pole (rearmost portion). **e** A fully flattened view of the cortical surface. The aura-related changes are localized wave segments. Note that in the flattened cortex was cut along the steep sulcus calcarine to avoid large area distortions induced by the flattening process. The *colored border to the left* is the cut edge that should be considered being connected such that the color match up as seen in **b** and **d**. Copyright (2001) National Academy of Sciences, USA [12]

The neural activity and subsequent changes in blood flow are closely coupled called neurovascular coupling. Not only the magnitude but also the spatial location of blood flow changes reflect neural activity, a fact that is used in noninvasive brain imaging techniques such as functional magnetic resonance imaging (fMRI). SD, however, is a pathological state in which this coupling is to some degree impaired, in particular evoking long lasting decreases phase (oligemia). To measure the spatial confinement of SD in human cortex during a migraine attack with fMRI, not merely hypermia, but also several other characteristic neurovascular events were used that resemble SD, among others [12]: the initial hyperemia with a characteristic duration followed by oligemia with recovery to baseline, the characteristic velocity of these events, and a concurrent recovery of stimulus driven activation (see Fig. 2).

While the pathological activity of neurons during SD is mainly characterized by a depressed state with literally no activity—hence the name, in the tissue ahead of SD, high-frequency activity and increased synaptic noise has been recorded [24]. The tissue surrounding the current location of SD is functionally connected through lateral neural networks with neurons that undergo seizure-like discharging at the rising front of SD. This provides an electrical signal transmission pathway several orders faster than SD, that is, in a first approximation an instantaneous connection. As firstly suggested by Wilkinson [25], the feed-forward and feedback cortical circuitry can also explain a more global hypermia by neural and synaptic activation in adjacent cortical areas, which might be mistaken as the area SD traverses, when only hypermia is used to estimate the spatial extend of SD in human cortex during a migraine attack with noninvasive imaging.

Increased neural activity and the resulting hypermia in an extended area surrounding SD can make the tissue that is yet to be recruited into the depressed SD state less susceptible to it and possible completely protect it from SD. Therefore, this mechanism could provide a neuroprotective mechanism that we mimic by the inhibitory mean field feedback. It is reasonable to assume that the larger SD spreads out, the large the increase in neural activity in the surrounding tissue and subsequent hypermia with neuroprotective effect. The coupling between increased neural activity and subsequent changes in cerebral blood flow has a significant time delay in the order of seconds, which we ignore for the sake of simplicity in our model.

## 3 Design of a Macroscopic Model for Migraine Aura

In this study, we are interested not only in the pattern forming mechanism of transient localized traveling waves, but also in the generic spatio-temporal properties of such waves. To this end, we design a canonical reaction-diffusion model (Sect. 3.1) augmented by an inhibitory mean-field feedback control (Sect. 3.2). The use of this model is motivated as described in the previous section. The phenomena our attention will be focused on in the next section are nucleaction, growth, and subsequent shrinking of the transient wave segments. To study statistical properties of these events, we must consider the set on initial conditions (see Sect. 3.3) over which statistical analysis are performed (Sect. 4), because these are features of transient dynamics.

### 3.1 Model Equations

The canonical model for excitable media are the well-known FitzHugh–Nagumo equations [26] with diffusion in the activator variable:

(1) $$\begin{array}{rl} \varepsilon \frac{\partial u}{\partial t} & =u-\frac{1}{3}u^{3}-v+\nabla^{2}u,\\ \frac{\partial v}{\partial t} & =u+\beta . \end{array}$$

The parameter *ε* separates the timescales of the dynamics of the activator *u* and the inhibitor *v*, and *ε* is taken to be small. In the present work, we use a value of ε=0.04. The parameter *β* is a threshold value which determines from which activator level on the inhibitor concentration is rising. The local dynamics of Eq. (1) (i.e., without the diffusion term) is oscillatory for |β|<1 and excitable for |β|>1. At |β|=1, the local dynamics undergo a supercritical Hopf-bifurcation. We choose a value of β=1.1 throughout this work. To integrate Eq. (1), we used a simulation based on spectral methods [27] and adaptive timestepping.

We define the (instantaneous) wave size as the area with activator level *u* over a certain threshold $u_{0}$:

(2) $$S(t):=\int \int H\left(u(x,y,t)-u_{0}\right)\;dx\;dy,$$

where *H* is the Heaviside function and we chose $u_{0}=0$.

**Fig. 3:**
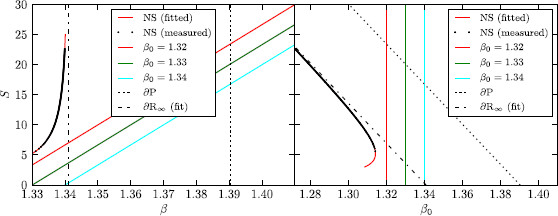
*Left*: The S−β plane with nucleation solution NS, propagation boundary *∂P*, rotor boundary $\partial R_{\infty }$, and control lines for the used values of $\beta_{0}$. *Right*: The $S-\beta_{0}$ plane with the same quantities

Equations (1) are a paradigmatic model of an excitable medium even beyond neuroscience [28]. They possess a stable homogeneous solution as well as stable excited states (pulses, spirals, or double spirals) cf. [29,30]. The boundary separating the basins of attraction of these types of solution is given by the stable manifold of the so-called “nucleation-solutions” (NS) whose stability is of saddle-type with one unstable direction; see Fig. 1. These nucleation-solutions are localized areas of excitation, which are traveling at uniform speed without changing shape. The size of these solutions, in the sense of Eq. (2), depends on the parameters *β* and *ε*. In Fig. 3, the size *S* of these nucleation solutions is plotted against *β*. This solution branch is also called *∂R* and the parameter value for which it diverges is called $\partial R_{\infty }$ or the “rotor boundary” (see next section). Of course, one could also use a measure different from *S* for visualizing the branch *∂R*, e.g., the propagation speed of the nucleation solution.

To obtain solutions lying on *∂R*, we used a pseudo-continuation procedure, which is described below.

Making the parameter *β* dependent on the wave size *S* adds a mean field control to the system.

(3) $$\beta =\beta (t)=\beta_{0}+K\cdot S(t),$$

where *K* and $\beta_{0}$ are control parameters.

This equation defines a straight line in (β−S)-space, we call the *control-line*. If the control line intersects *∂R* (in (β−S)-space), the point of intersection with higher *S* is stabilized, cf. [31]. This is visualized in a movie; see Supplementary Material Video 1. This can be understood intuitively, as the stable manifold of a point on *∂R* separates the attraction basin of the homogeneous solution for which *S* shrinks until S=0 and the attraction basin of rotating spirals with growing S→∞. Imagine the system to be on *∂R*, that is, showing a nucleation solution as discussed above. If the current state is perturbed to have slightly smaller *S*, in the uncontrolled system we would have entered the attraction basin of the homogeneous solution (cf. Fig. 3). The control however forces the system to stay on the line defined by Eq. (3). If this line intersects *∂R*, a slightly smaller *S* makes the control adjust the value of *β* to smaller values, taking the system into the attraction basin of the spiral waves, where *S* will grow. The same process happens with different signs if the current state is perturbed to have slightly smaller *S* thus in effect stabilizing the (one unstable direction of) the nucleation solution.

The aim of the present work is to shed light on the transient behavior, occurring when the control line, Eq. (3) is close to *∂R* but does not intersect it as the ones depicted in Fig. 3.

To account for the imprecision in *∂R* of the simulation and the exact *∂R*, we measured the *∂R* in our simulation using a pseudo-continuation procedure. For this, we set the control such that it intersects *∂R*, and thus stabilize an otherwise unstable solution on it. Letting the simulation run until the system has stopped fluctuating, saving the (β,S)-pair, changing the control slightly and doing things over yields points of *∂R* in our system. From this measured *∂R* and the propagation boundary *∂P*, inferred from continuation in 1D, we chose 3 suitable control lines which were used for simulations in this work:

(4) $$\begin{array}{rl} K & =0.003,\\ \beta_{0} & \in \{1.32,1.33,1.34\}. \end{array}$$

With this procedure, it is not possible to obtain (β−S)-pairs below a certain value of *S*. The reason is that the system (with mean-field control) is undergoing a saddle-node bifurcation as visualized in the right of Fig. 3. For the purpose of visualization for this saddle-node bifurcation, we fitted the locus of the measured branch of nucleation solutions (in β−S space) to a function of the form $\beta =a+\frac{b}{cS+S^{2}}$ (where a,b, and *c* are the fit parameters). We have not only used this function for visualizing the saddle-node bifurcation, but we also deduced an approximate *β* value for the rotor boundary $\partial R_{\infty }$ by letting S→∞.

### 3.2 Effect of Mean Field Inhibitory Feedback Control

The diversity of the behavior of traveling waves in two spatial dimensions was studied in canonical models depending on the two generic parameters *β* and *ε* in Eq. (1), which determine the parameter plane of excitability without mean field inhibitory feedback [32]. In those media, patterns of discontinuous (open ends) and spiral-shaped waves are used to probe excitability. These spiral patterns are closely related to the discontinuous, localized transient waves we propose in our model. In fact, the effect of mean field inhibitory feedback control can best be understood, if we compare these patterns in models with and without this control.

In the design of our model, we make use of the fact that in a model without mean field inhibitory feedback control spiral waves do not curl-in anymore, but become half plane waves at a low critical excitability, called the rotor boundary $\partial R_{\infty }$[33,34]. Beyond the rotor boundary lies the subexcitable regime in which discontinuous waves start to retract at their open ends and any discontinuous wave is transient and will eventually disappear (see Videos 2 and 3). In other words, spirals do not exist beyond $\partial R_{\infty }$. The boundary $\partial R_{\infty }$ marks a saddle-node bifurcation at which discontinuous spiral waves collide with their corresponding nucleation solution. This leads to the key idea of our model, namely to introduce mean field inhibitory feedback control. A linear mean field feedback control moves this saddle-node bifurcation toward distinct localized wave segments with a characteristic form (shape, size) and behind this bifurcation these waves become transient objects; see Fig. 1, Fig. 3, and Video 4.

Before we further consider the effect of mean field inhibitory feedback control, we have to describe the behavior of continuous waves (closed wave fronts without open ends) when excitability is decreased, e.g., by increasing *β*, without mean field inhibitory feedback control. This will be important if we want to understand the fate of any solution, discontinuous or not, under mean field feedback control. Unbroken plane waves propagate persistently even if the parameters are chosen in the subexcitable regime until *β* reaches a value called the propagation boundary *∂P*. At this boundary, the medium’s excitability becomes too weak for continuous plane waves to propagate persistently. The boundary *∂P* in parameter space marks also a saddle-node bifurcation at which a planar traveling wave solution collides with its corresponding nucleation solution. Note, that the planar wave is essentially a pulse solution in 1D and the nucleation solution in 1D is called the slow wave [35].

In Fig. 3(left), both the rotor boundary $\partial R_{\infty }$ and the propagation boundary *∂P* are shown in a bifurcation diagram for the excitable medium described by Eqs. (1). We chose *β* as the bifurcation parameter and follow (see previous section) the branch *∂R* of the unstable nucleation solution (NS) whose stable manifold separates the basins of attraction of the homogeneous state and a spiral wave (with two counter-rotating open ends). The unstable manifold of NS consists of the two heteroclinic connections, one to the stable homogeneous state and the other to the traveling wave solution (see Fig. 1). The order parameter on the ordinate in Fig. 3 is the surface area *S* inside the isoclines at $u=u_{0}=0$ of the traveling wave solutions; see Eq. (2).

The mean field control that we introduce by Eqs. (2)–(3) establishes a linear feedback signal of the wave size *S* to the threshold *β*. With this linear relation, we introduce two new parameters, the coupling constant *K* and $\beta_{0}$, the threshold parameter for the medium without an excited state (S=0). Note that the parameter $\beta_{0}$ can be also seen as the sum of two threshold values, the former *β* in Eq. (1) and an offset coming from the new control scheme. While the introduction of the control introduces two new parameters $\beta_{0}$ and *K*, at the same time *β* becomes dependent upon the control, so that we have a total of three free parameters.

We chose $\beta_{0}$ as the new bifurcation parameter in the bifurcation diagram for the completed reaction-diffusion model with mean field coupling described by Eqs. (1)–(3); see Fig. 3(right). This diagram is a sheared version of the one without mean field coupling in Fig. 3(left). While it is a trivial fact that the linear relation in Eq. (3) describes an affine shear of the axes (β,S) of the bifurcation diagram in a to the new axes $\left(\beta_{0},S\right)$ in b, the fact that the branch *∂R* of the nucleation solutions can be mapped this way is not. Firstly, this relies on the way we introduce the feedback term. It just adds a constant value to the old bifurcation parameter *β*, if the solution under consideration is stationary. Therefore, any stationary solution must exist in both diagrams being just sheared branches. The same still holds true for traveling wave solutions that are stationary in some appropriate comoving frame, for instance, ξ=x−ct with speed *c*. However, not much can be said about the stability of such solutions, when we introduce the mean field feedback term.

The branch *∂R* of the formerly unstable nucleation solutions NS (Fig. 3(left)) folds in Fig. 3(right) such that two solutions coincide for a given value of $\beta_{0}$ until they collide and annihilate each other at a finite value of S≈5.5 for K=0.003. For the fixed value of K=0.003, the upper branch consists of stable traveling wave solutions in the shape of a wave segment, while the lower branch belongs to the corresponding nucleation solutions of these wave segments, as schematically shown in Fig. 1b. The fact that the upper branch is stable was confirmed by numerical simulations (cf. Sect. 3.1 and Video 1). Larger *K*, that is, a less steep control line in Fig. 3(left) can be seen as a “harder” control, because a small given change in *S* leads to larger variations in the effective parameter *β*. As a consequence, it is difficult to stabilize lower part of the branch corresponding to small traveling wave segments in numerical simulations by means of this control.

The choice of the parameter regime given by Eq. (4), which shows only transient localized waves for this model and leads to a globally stable homogeneous state as the only attractor, is straightforward given the branch *∂R*. In this sense, we designed the model to exhibit transient localized waves due to a bottleneck—or ghost behavior—after the saddle-node bifurcation.

### 3.3 Initial Conditions

We need an appropriate sampling of initial conditions for Eqs. (1)–(3), ideally being equidistantly sampled in some distribution. The set of all initial conditions for this system does not—to our knowledge—carry a helpful mathematical structure which allows us to achieve this aim easily. In order to attack this problem, we turned to the physiological motivation of the chosen model explained in Sect. 2.

**Fig. 4:**
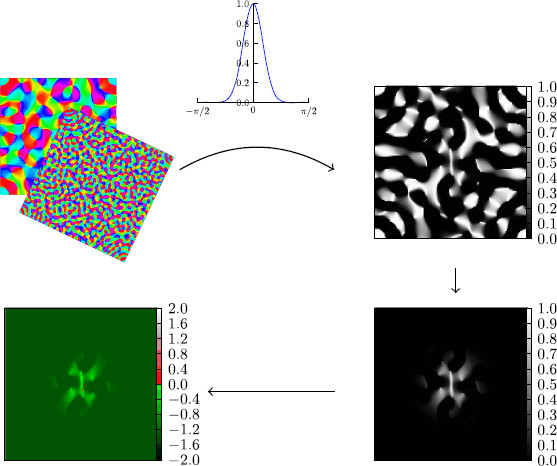
To construct initial conditions from artificially generated pinwheel maps, we first took such a pinwheel map with a certain *scaling* (*upper left*), then we chose a selection of excited orientations by means of a Gaussian. The width of the Gaussian gives the selection *depth* (*upper right*). After that, we masked the result spatially with another Gaussian distribution that is radially symmetric (*lower right*). The width of this Gaussian gives the third parameter, the *size* of the pattern. Finally the result is scaled, giving rise to the fourth parameter, we called the *excess* and added to the activator variable in the homogeneous state (*lower left*). The inhibitor variable is put into the homogeneous state

A set of initial conditions should naturally reflect plausible spatial perturbations of the homogeneous steady state of the cortex. This can be achieved by defining localized but spatially structured activity states on large scales of the order of millimeters. Such pattern are obtained from cortical feature maps (see Fig. 4) by sampling three parameters (*scaling*, *depth*, and *size*) that define patches of lateral coupling in theses maps. A fourth parameter (*excess*) determines the amplitude of the perturbation. In the following, we first describe the rational behind using a cortical feature map and then the sampling.

#### 3.3.1 Rational to Use Cortical Feature Map

We focus on a cortical feature map in the primary visual cortex (V1) called the pinwheel map. V1 is located at the occipital pole of the cerebral cortex and is the first region to process visual information from the eyes. Migraine aura symptoms often start there or nearby where similar feature maps exist.

In V1, neurons within vertical columns (through the cortical layers) represent by their activity patterns edges, elongated contours, and whole textures “seen” in the visual field. This representation has a distinct periodically microstructured pattern: the pinwheel map. Neurons preferentially fire for edges with a given orientation and the preference changes continuously as a function of cortical location, except at singularities, the pinwheel centers, where the all the different orientations meet [36,37].

Iso-orientation domains form continuous bands or patches around pinwheels and, on average, a region of about 1 mm^2^ (hypercolumn) will contain all possible orientation preferences. This topographical arrangement allows one hypercolumn to analyze all orientations coming from a small area in the visual field, but as a consequence, the cortical representation of continuous contours in the visual field is depicted in a patchy, discontinuous fashion [38]. In general, spatially separated elements are bound together by short- and long-range lateral connections. While the strength of the local short-range connection within one hypercolum is a graded function of cortical distance, mostly independent of relative orientation [39], long-range connections over several hypercolumns connect only iso-orientation domains of similar orientation preference [40,41]. Even nearby regions, which are directly excitatory connected, have an inhibitory component through local inhibitory interneurons and this is likely be used to analyze angular visual features such as corners or T junctions [39].

**Fig. 5:**
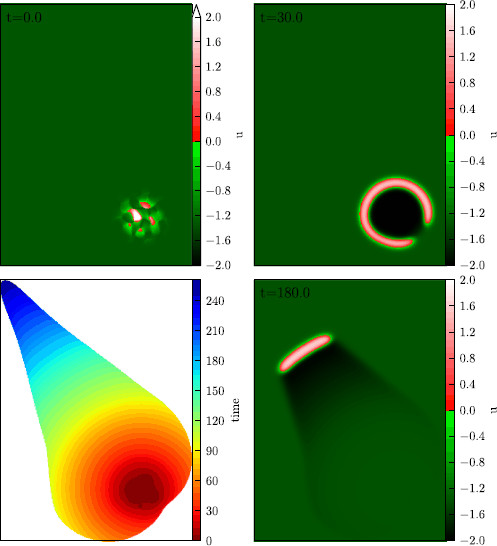
An example of a transient solution. Initial conditions, i.e., activator concentration *u* at 0 sec (*upper left*), snapshot of activator-concentration *u* after 30 sec (*upper right*), after 180 sec (*lower right*). Time of passing through threshold value $u_{0}=0$ from below the first time, i.e., passing of the wave front (*lower left*)

Given the arguments above, we can now obtain localized yet spatially structured activity states on the scale we aim for as initial conditions by using iso-orientation domains that form continuous patches around pinwheels and extend in a discontinuous fashion over larger areas. In these patches, neural activity can get into a critical mode, like neural avalanches [42] that would locally perturb the ionic homeostasis as exemplary shown in Fig. 5 (lower left).

#### 3.3.2 Sampling of Patterns in Cortical Feature Maps

In [43], the authors analyzed the design principles that lie behind the columnar organization of the visual cortex. The precise design principles of this cortical organization is governed by an annulus-like spectral structure in Fourier domain [36,43], which is determined by mainly one parameter (*scaling*), that is, the annulus width. The parameter *depth* reflects the tuning properties of orientation preference or we can also interpret this as the range of orientation angles that we consider within the iso-orientation domain. The third parameter reflects the distance long-range coupling ranges before it significantly attenuates.

These design principles can be exploited and a procedure can be designed to construct maps with the same properties. The constructed maps come very close to the maps found in brains of macaque monkeys (see [43] and references therein).

To construct initial conditions from these maps we used a procedure that uses four control parameters and is visualized in Fig. 4. The details are as follows: A pinwheel map is a function that maps our two-dimensional plane to the interval (−π/2,π/2]. We construct such a map using the procedure in [43]. During construction, we can choose the *scaling* of the map. This is our first parameter. After constructing this map, by means of a Gaussian, we choose a range of orientations that is excited. Mathematically speaking, this is the concatenation of the Gaussian distribution with the pinwheel map. This gives the next parameter, namely the width of the Gaussian that selects the angles, we call that parameter the *depth*. The next step is to constrain the generated pattern spatially by multiplication with another Gaussian, which is defined on the plane *P* and chosen to be rotationally symmetric. The width of this Gaussian gives rise to the third parameter, the *size* of the pattern. Finally, we multiply the pattern by a certain amplitude, which is chosen such that the integral of the pattern over the plane gives a chosen number, which constitutes the fourth parameter, we called the *excess*.

Finally, initial conditions are generated by setting the plane to the (stable) homogeneous state and then adding the generated pattern, which represents increased activity like in neural avalanche, to the activator variable *u*, which represents the ionic imbalance most notably the extracellular potassium concentration.

In a first run, we scanned the space spanned by the four parameters coarsely. We used the marginal distributions of the number of solutions with an excitation duration (ED) >0 with respect to the parameters to decide how densely to sample the parameter space in the final run.

## 4 Statistical Properties of Transient Localized Waves

To explore the typical transient patterns that the system described by Eqs. (1)–(3) generates, we want to know how the system responds to the initial conditions as described before. In characterizing the transient solutions, the same problem we had to obtain equally space initial conditions arises when appropriate characteristic parameters for the solutions have to be defined.

To explain the three parameters, we have chosen to characterize the solutions and why they suit this problem, it is helpful to have a look at the lower left part of Fig. 5, in which an example solution is displayed. The first parameter we chose is the maximal area in which such a solution has activator concentration over a certain threshold level at one instant of time, termed maximal instantaneous area (MIA). The threshold level is taken to be u=0, although this is the same threshold as used for $u_{0}$ to define *S*, this is rather convenience than necessity. The second parameter is the total area that has experienced an activator concentration above this level at some time during the course of the solution, termed total affected area (TAA). The third parameter is the time, during which the area of activator concentration above threshold is nonzero, termed the excitation duration (ED). Of course, the exact value of all these parameters for one single solution depends on the choice of threshold. For once, the threshold value has to be chosen such that after the activator concentration has fallen below it; no secondary excitation will be generated.

The example solution depicted in Fig. 5 is a comparatively long lived solution (Video 4). It starts out very symmetrically (circular) shaped, at one instant of time it breaks open into a discontinuous wave and a shape of the front develops, which is similar to that of a particle-like wave but because of the chosen control parameters, it shrinks in time and vanishes in the end. Because at the point when the circular front breaks open, a comparatively large area is affected, it takes some time until it vanishes and the resulting TAA is relatively large. So this example solution has large ED, large MIA, and large TAA. If the circular front had not broken open at all, the control would have made the threshold value very large and the solution would have collapsed very quickly because of the propagation boundary *∂P*, such that the ED and the TAA would have been short, whereas the MIA would have been large. Other prototypical courses of solutions take place for instance when the initial conditions affect the activator over a larger area, but only in the middle of the area; the value is high enough to start a solution. In the surrounding area, the activator level is not high enough for that but the increased activator concentration leads to a rise in inhibitor concentration until the time the front reaches those parts and as a consequence, the solution vanishes early, having small ED, small TAA, and small MIA as a consequence.

**Fig. 6:**
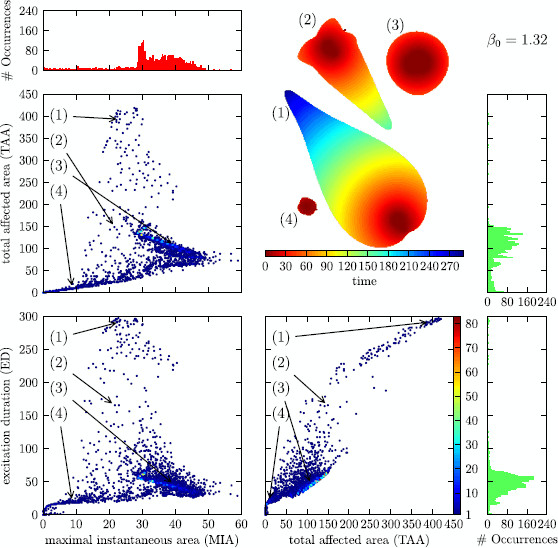
Distribution of solutions for the control close to *∂R*

**Fig. 7:**
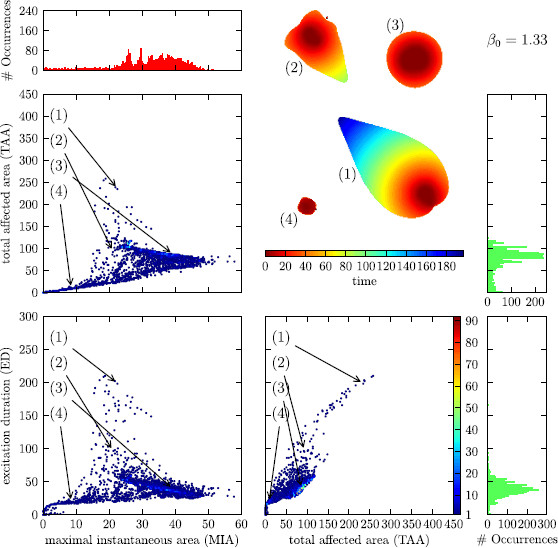
Distribution of solutions for the control line at an intermediate distance to *∂R*

**Fig. 8:**
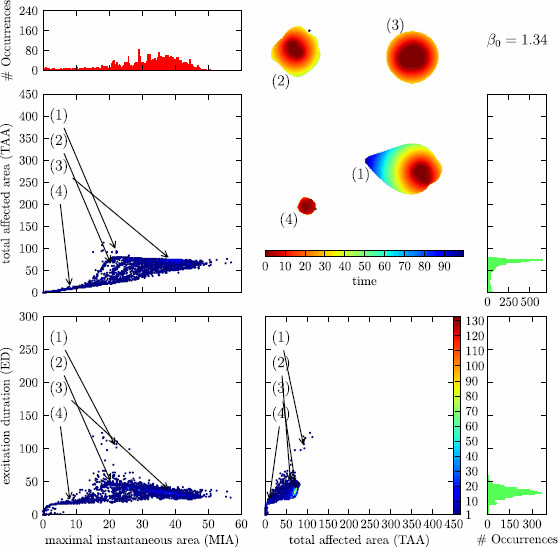
Distribution of solutions for the control far away from *∂R*

We did the simulation for three different adjustments of the control force, successively going farther and farther away from the bifurcation point. Each of these simulations were started using 8,000 initial conditions generated in a manner that is described in Sect. 3. Each of these initial conditions resulted in a solution that was classified according to the three parameters mentioned above. The solutions that did not result in any excitation at all (ED = MIA = TAA = 0) were discarded. The density plots according to the classification parameters are shown in Figs. 6, 7, 8. First of all, though the distribution of the solutions varies significantly, the number of solutions that represent an excitation hardly varies at all (4171 for small, 4183 for intermediate, 4182 for large distance from the bifurcation point), the symmetric difference between the sets of initial conditions that lead to an excitation contains between 5 and 19 solutions. (The symmetric difference of two sets *A* and *B* is defined as A△B:=(A∪B)∖(A∩B). It is the set of all elements that are contained in one and only one of *A* or *B*.) From this, we can also deduce that the set of initial conditions that lead to an excitation does not significantly depend on the choice of control parameters.

When looking at Fig. 6, one notices a clustering of the solutions in certain regions of the classification parameters. In the section that depicts the TAA against the MIA, we notice three coarse clusters. Cluster I, the largest with high MIA and comparatively low TAA; cluster II, one that is less populated with low MIA and low TAA; and cluster III, one that is very sparsely populated with intermediate MIA and high TAA. The boundaries between these clusters are not very sharp. One could think that a solution that affects an overall large area (high TAA) will also affect a large area at one instant of time (high MIA). From looking at the mentioned clusters, one sees that this is not the case, the solutions with the highest MIA have all comparatively low TAA (clusters I and II) and the ones that have a high TAA only achieve an intermediate MIA (cluster III).

A partial explanation for this can be read off from the depiction of TAA against ED. All solutions that have a large TAA are also solutions that have a large ED, i.e., cluster III is distinct also in this plane. More than that, the dependence seems to be almost linearly. This is reminiscent of the localized particle-like wave solutions. For these, the area that is affected grows linearly in time because the area that these solutions occupy at one instant of time is constant.

The two clusters I and II that we observed merge to one in this plane of projection because they differ only very little in ED. This can also be noted when comparing the planes MIA vs. TAA and MIA vs. ED; also here, the cluster III with high TAA translates to a cluster with high ED and the cluster I with high MIA and comparatively low TAA moves closer to cluster II with both low ED and MIA.

When varying the $\beta_{0}$ parameter of the control force, the distribution of solutions in MIA–TAA–ED-space changes drastically. Upon raising the $\beta_{0}$ parameter from $\beta_{0}=1.32$ over $\beta_{0}=1.33$ to $\beta_{0}=1.34$, the system is put more and more into the subexcitable regime and the solutions are less and less affected by the ghost behavior (saddle-node bifurcation); see Fig. 3. This is noticeable by observing that the cluster with high TAA/high ED becomes less pronounced and vanishes almost completely for $\beta_{0}=1.34$. This can be understood as an interplay between the mean value of MIA in cluster III at about 25 and *S* at the propagation boundary (at *∂P*, S≈24, S≈20.75, and S≈17.5 for $\beta_{0}=1.32$, $\beta_{0}=1.33$, and $\beta_{0}=1.34$, respectively). For the control line farthest away from the saddle-node bifurcation ($\beta_{0}=1.34$), *∂P* is below even the smallest values of MIA in cluster III. Note that the value of *S* at the ghost is about 6, well below the propagation boundary. Also, the other two clusters merge though there still exist solutions with high and with low MIA, but the transition is much more fuzzy than it was before.

In Figs. 6, 7, and 8, we have included a little “bestiary” to illustrate the typical courses of solutions in the respective clusters and their change upon varying the parameter $\beta_{0}$, the initial conditions for solutions 1–4 in these figures are always the same. From this arbitrarily chosen selection, we see that the MIA of each solution hardly changes between the $\beta_{0}$ values, whereas the change of TAA and ED always go hand in hand and—depending on the cluster—can be up to four-fold for the chosen range of $\beta_{0}$.

One could argue that the formation of clusters is an artefact of the choice of initial conditions. There is no simple answer to this. As mentioned, it is not possible to examine the complete set of initial conditions. Neither does this set carry a helpful structure which would allow a sensible “equidistant” sampling. This is the reason why we made the mentioned choice of initial conditions. For testing purposes, we also tried different schemes for the generation of initial conditions and found the same distribution of clusters qualitatively.

**Fig. 9:**
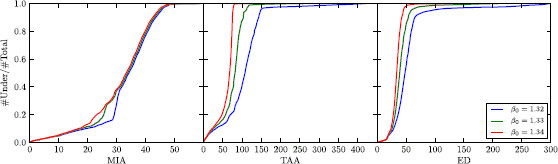
Cumulative distribution functions for the different classification parameters

In Fig. 9, we have plotted the cumulative distribution functions for the three classification parameters and the three choices of mean field control. From this picture, we see that the distribution of the MIA is hardly influenced by the choice of control. This is very different for TAA and ED. For the TAA, for example, there are values (around 75), where for one choice of control the majority of solutions is below and for another choice the majority is above. For example, the fraction of values below TAA = 80 is 0.995 for $\beta_{0}=1.34$ and 0.216 for $\beta_{0}=1.32$. Also, we see that the cumulative distribution function for the TAA converges to 1 much slower, the closer the control is to the saddle-node bifurcation. This means that more solutions with high TAA exist for these choices of control.

## 5 Discussion

In this section, we discuss three subjects related to the intended application to migraine pathophysiology. Firstly, we start with the discussion about the applicability of this canonical model. Secondly, the possible congruence between the prevalence of migraine subforms with the statistical properties of the wave patterns we observed is discussed. Thirdly, we end with a brief outlook on novel therapeutic approaches in episodic migraine based on the here suggested pattern forming processes.

### 5.1 Canonical Model and Free Parameters for Weakly Excitable Media

Central to our approach is the localization resp. spatial confinement of the transient traveling waves. Reaction-diffusion waves would engulf all of the medium, if formed in a two-variable system with only one activator and one inhibitor with the system’s parameters in the appropriate regime. In contrast, localized traveling waves indicate a demand-controlled excitability. Similar ideas to obtain localized traveling, though not transient, waves have been introduced in various contexts, for instance, an integral negative feedback or a third, fast diffusing inhibitory component for moving spots in semiconductor materials, gas discharge phenomena, and chemical systems [31,44-46]. Furthermore, in neural field models [47], localized two-dimensional bumps are studied [48-50] in integrodifferential equations (without diffusion) in the context, for example, of memory formation [51]. Localized structures have also been discussed in the context of cortical spreading depression (SD) in migraine before, in particular a model with narrowly tuned parameters that shows transient waves [13,52,53] and a model with mean field feedback control that allows for localized waves [30]. But it is for the first time now that a model is presented in which wave phenomena occur that are both localized and transient, so that a variety of new questions that are controversially discussed in migraine research [18,54-56] can be addressed. A central question is of course, which level of detail a model of SD needs to investigate localization of SD and the transient response properties.

Physiological detailed models of SD are given by conductance-ion-based models with 9 to 29 dynamical variables in various (∼200) electrically coupled neural compartments [57-59]. They usually do not include lateral space—the compartments extend in the vertical direction to model the apical dendritic tree, that is, these models are not spatially extended to describe an excitable medium. In fact, this lateral extension is far from straightforward. Naively adding diffusion to the extracellular dynamical ion concentrations is possible but does not reflect the necessary detail that needs to be considered to take the spatial continuum limit. Neural field models, for example, describe this limit [47]. The first part of the global inhibitory feedback by neurovascular coupling, i.e., the fast spread of neural hyperactivity that initiates hypermia—as we suggested in this study—can be modeled by neural fields. The second part of this neurovascular coupling, the actual feedback signal needs also some physiological detail. This cannot easily be incorporated because neural field models are rate-based or activity-based while the detailed SD models [57-59] start with a conductance-based Hodgkin–Huxley approach and include ion-based dynamics but do not describe the dynamics on a rate-based foundation.

Even if a SD detailed tissue-model, that is, an excitable medium with the same physiological detail as in the single cell models of SD [57-59], were available, it would require enormous computer capabilities to calculate from the activity of single cells on the scale of milliseconds the macroscopic patterns on the spatial scale of several centimeters and on a temporal scale of up to one hour. Quite apart from the fact that five orders of magnitude in both the spatial and temporal scales suggest that the macroscopic phenomena require their own level of description in some form of effective medium theory.

We suggested that the primary objective in research relating SD to migraine should be to obtain a measure of the noxious signatures that are transmitted into the meninges during SD [60,61]. Canonical reaction-diffusion models seem to be at least one way to approach this objective. A further advantage of such canonical models lies in the fact that they allow insight in the phase space structure of the whole class of models they represent, as schematically shown in Fig. 1. Therefore, in the following, we will argue in which sense our model is canonical for the problem we attack.

Generally speaking, an excitable medium is a spatially extended system with a stable homogeneous steady state being the quiescent state and one or many excited states that develop after a sufficient perturbation from the quiescent state (Fig. 1a). The excited states are traveling wave solutions that propagate with a stable profile of permanent shape (possibly with some temporal modulation, such as breathing or meandering). To study generic features of an excitable medium, the simulations are often carried out in the reaction-diffusion system given by Eqs. (1), the popular FitzHugh–Nagumo kinetics. Originally, the FitzHugh–Nagumo kinetics were a caricature of the electrophysiological properties of excitable membranes [62,63], but these equations with D=0 became a canonical model of *local* excitability of type II (based on Hopf bifurcation, either supercritical with subsequent extremely fast transition to a large amplitude limit cycle, named canard explosion [64], or subcritical [65]). For D≠0, the FitzHugh–Nagumo kinetics became also a canonical model for *spatial* excitability [32]. Sometimes diffusion in the second inhibitory species is included, which we do not consider here. Because we investigate transient behavior originating from a high threshold regime (toward weak excitability), the classification of local excitability in types I and II (based on the transition at vanishing threshold, i.e., into the oscillatory regime) is not relevant. Furthermore, it is not clear whether this classification carries over in a meaningful way to the dynamics of spatially extended systems.

We consider the set of Eqs. (1) as canonical for two reasons. First, because the *u* (activator) equation of Eq. (1) has the simplest polynomial form of bistability. Note that for this reason this activator equation was originally suggested by Hodgkin and Huxley as the first mathematical model of the potassium dynamics in SD. It was published by Grafstein, who also provided experimental data supporting such a simple reaction-diffusion scheme for the front dynamics in SD [66]. Second, the inhibitor equation of Eq. (1) has a linear rate function, in fact, the rate function is only a function of the activator *u*. This is the simplest inhibitor dynamics needed for pulse propagation. By neglecting an additional linear term −γv in the inhibitor rate function, we limit the origin of excitability to the case of a supercritical Hopf bifurcation with subsequent canard explosion and avoid the bistable regime that exits in the subcritical case. The subcritical Hopf bifurcation occurs only in a narrow regime when *γ* is close to 1 and *β* close to 0. We have tested some simulations with γ=0.5 with similar results.

As a consequence of our assumptions about the model being in this canonical form, only two free parameters exist, *β* which is associated with the threshold and the *ε*, the time scale separation of activator and inhibitor dynamics. Of course, the choice of parameters can be quite different, a common choice is *α* in the cubic rate function f(u)=u(u−α)(u−α) but there are only two free parameters or two equivalent groups of parameters. So, there are the same bifurcations in the parameter planes (ε,β) or (ε,α), but to map the dynamics between equivalent groups of parameters might involve changes in time, space, and concentrations scales.

In particular, the question of how the incidence of MA is reflected in the distance to the saddle-node bifurcation, involves a measure on the parameter space whether is (ε,β), (ε,α), or any other parameter plane. We have previously suggested to get such measures from pharmacokinetic-pharmacodynamic models [53].

### 5.2 Application to Migraine Pathophysiology

We suggest a qualitative congruence between the prevalence of MO and MA with the statistical properties we found in the transient response properties. We do not suggest that all migraine attacks are related to SD nor that pain formation in MA is exclusively caused by SD. Rather that SD is one pathway of pain formation in both symptom-based subtypes MO and MA. We refer to this pathway as the “spreading depression”-theory of migraine [17]. The “migraine generator”-theory (MG) is for various reasons not less plausible [54]. It assumes a dysfunction in a central pattern generator in the brainstem that modulates the perception of pain. Some of the seemingly conflicting and controversially discussed evidence is probably resolved when one considered the basis of the classification of migraine subforms. We currently have a symptom-based classification for migraine with possibly overlapping etiologies for individual subforms. In the light of an etiology-based classification with possibly overlapping symptoms the conflicts seem less puzzling to us. To further resolve this, we investigated the interplay of SD and MG and suggested to unify these approaches within a network theory [61].

In the remainder of this section, we focus first on migraine pain and then on the migraine aura.

#### 5.2.1 Migraine Pain

**Fig. 10:**
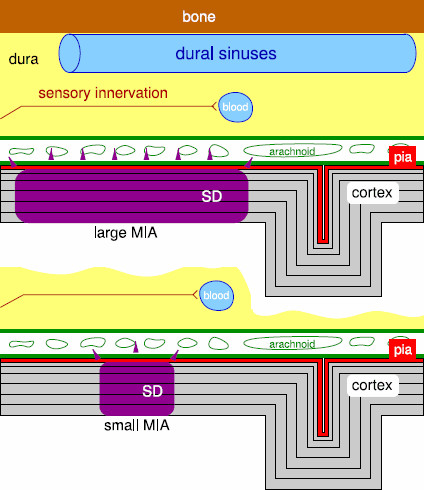
Schematic representation of cross section of cortex, meninges, and skull. The leptomeninges refer to the pia mater and arachnoid membrane. SD releases noxious substances with increased blood flow thought to diffuse outward. Activation of pain pathways can depend on MIA

The cortex is not pain sensitive. Therefore, SD in the cortex cannot explain the headache phase in migraine. There are detailed investigations how SD in the cortex can cause pain via pain sensitive intracranial tissues and subsequent activation in the trigeminal nucleus caudalis in the brainstem [67,68], but cf. [69,70]. The qualitative congruence between the prevalence of MO and MA with the statistical properties we found in the transient response properties is based on the following assumption on the geometrical layout of the cortex and the pain sensitive cranial tissues (Fig. 10): In the initial phase of cortical SD, with increased blood flow (hyperemic phase), a local release of noxious substances (ATP, glutamate, K^+^, H^+^) are thought to diffuse outward in the direction perpendicular to the cortex into “the leptomeninges resulting in activation of pial nociceptors, local neurogenic inflammation, and the persistent activation of dural nociceptors, which triggers the migraine headache” [71], but for issues concerning the blood brain barrier system cf. [72].

If diffusion vertical to the affected cortical area is critical, size and shape of this area should play a critical role; see Fig. 10. This suggests that SD waves activate nociceptive mechanisms dependent upon a sufficiently large instantaneously affected cortical area, i.e., large MIA and, as stated before, the primary objective should be to obtain a measure of the noxious signatures that are transmitted into the meninges during SD.

#### 5.2.2 Migraine Aura

The aura phase, on the other hand, must clearly correlate with long duration of SD and a large enough cortical surface area being affected during the course of SD to notice neurological deficits. In particular, because the very noticeable visual symptoms often start where the cortical magnification factor is large, so that only if they move into regions of lower magnification they get significantly magnified by the reversed topographic mapping [52]. The seemingly contested notion of MO (migraine without aura) with silent aura can also be resolved when the spatiotemporal development of SD is taken into account.

**Fig. 11:**
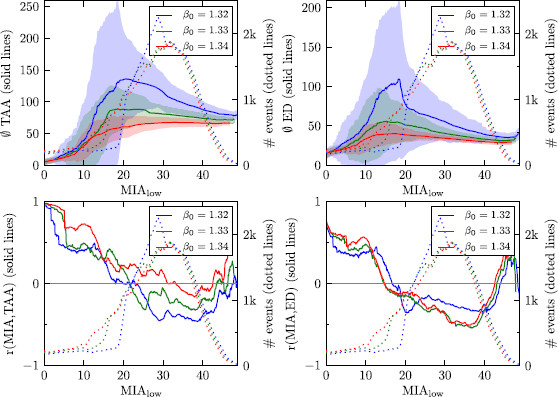
Statistical analysis of output data. For all *four pictures*, we took all data points with MIA in the interval $\left[{\mathrm{MIA}}_{\mathrm{low}},{\mathrm{MIA}}_{\mathrm{low}}+10\right]$ (“sliding window”) and analyzed the connection with TAA (*left column*) and ED (*right column*). In the *upper row*, the average value is plotted with *solid lines*; the area of one standard deviation around this value is *shaded*. In the *lower row*, the correlation coefficient between MIA and the respective quantity is plotted. In all plots, the *dotted lines* indicates the number of events for the respective interval with MIA_low_

The connection between MIA and TAA as well as MIA and ED in our model is shown in Fig. 11. It shows that in the range of high MIA the average values for TAA and ED are becoming smaller. From Fig. 11, we can also read off that the range with the most events is in the regime of relatively high MIA (around 30) and significantly after the peak of ED resp. TAA. Moreover, in the range with most events, the correlation coefficient r(MIA,ED) is always negative and the correlation coefficient r(MIA,TAA) is mostly negative. All these effects are stronger, the closer the control line is located to the saddle-node bifurcation.

From these statistical correlations between MIA and TAA resp. ED and the distribution of the number of events, one could speculate that cases of MA are more rare and the quality of the headache in these cases might be less severe. This is exactly what has been reported in the medical literature [73].

While the number of events with high ED and high TAA is influenced by the distance of the control line to the saddle node bifurcation, the number of events with high MIA is much lesser affected. So, in a way, the distance to the saddle node bifurcation controls the prevalence of MA in our model, while the prevalence of MO is not much affected.

### 5.3 Model-Based Control by Neuromodulation

We briefly discuss model-based control and means by which neuromodulation techniques may affect pathways of pain formation and the aura phase.

**Fig. 12:**
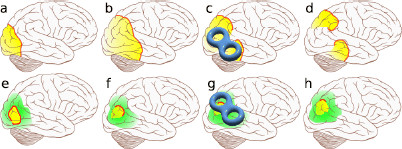
Development of SD and model-based control. SD based on a two-variable reaction-diffusion mechanism engulfs all the densely packed excitable neurons in the cortex (*top row*). The activator concentration is in *red*, the inhibitor in *yellow* in this schematic illustration. Long-range inhibitory feedback (*green*) is a well-established pattern formation mechanism to confine the spread (*bottom row*). The predicted emerging transient patterns offer a model-based analysis of phase-depended stimulation protocols. In the classical paradigm, applying noninvasive neuromodulation devices, which may succeed to block SD locally (**c**), would result in a reentrant pattern (**d**). In the new paradigm of localized SD waves that we suggest, a phase-dependent stimulation protocol might target intelligently with noise the bottle-neck passage (**g**) (see main text), while a different protocol might be considered during the initial nucleation phase (**e**)

The emerging transient patterns and their classification according to size and duration offer a model-based analysis of phase-dependent stimulation protocols for noninvasive neuromodulation devices, e.g., utilizing transcranial magnetic stimulation (TMS) [74], to intelligently target migraine. For instance, noise is a very effective method to drive the system back into the homogeneous steady state more quickly; see Fig. 12. In general, responses of nonlinear systems to noise applied when the system is just before or past a saddle-node bifurcation are well studied. Before the saddle-node on limit cycle bifurcation, the phenomenon of coherence resonance (CR) describes that a certain amount of noise makes responses most coherent [75]. Behind the saddle-node bifurcation on a limit cycle, the time the flows spend in the bottleneck region of the ghost is shortened [76]. However, noise would, according to our model, mainly positively affect ED and TAA, that is, the aura, while it could even worsen the headache, if applied early during the nucleation and growth process. Therefore, TMS with noise stimulation protocols, which are currently investigated, should be applied only some time after first noticing aura symptoms.

Headaches are not generally considered appropriate for invasive neurosurgical therapy, but when all else fails—preventives, abortives, and pain management—invasive brain stimulation techniques are also considered, e.g., occipital nerve stimulation (ONS) [77,78]. So model-based control will become increasingly important. Also, the importance of modeling related epileptic seizure dynamics as spatiotemporal transient patterns has been suggested in a recent paper [79]. Model-based control of Parkinson’s disease, is already considered, yet Schiff remarks quite correctly [80]: “It seems incredible that the tremendous body of skill and knowledge of model-based control engineering has had so little impact on modern medicine. The timing is now propitious to propose fusing control theory with neural stimulation for the treatment of dynamical brain disease.”

We suggest to consider migraine as a dynamical disease that could benefit from model-based control therapies.

## Electronic Supplementary Material

## Competing Interests

The authors declare no conflicts of interest.

## Authors’ Contributions

MAD conceived the model and its application to migraine aura. TMI implemented the simulation. MAD and TMI analysed the mathematical model, interpreted the results, wrote the paper, and read and approved the final manuscript.

## Supplementary Material


Additional file 1
Transient localized wave patterns and their application to migraine
Click here for file

Additional file 2
Transient localized wave patterns and their application to migraine
Click here for file

Additional file 3
Transient localized wave patterns and their application to migraine
Click here for file

Additional file 4
Transient localized wave patterns and their application to migraine
Click here for file
